# Making the most of methylation

**DOI:** 10.7554/eLife.01387

**Published:** 2013-09-03

**Authors:** Michiel Vermeulen

**Affiliations:** 1 **Michiel Vermeulen** is at the Department of Molecular Cancer Research, University Medical Center Utrecht, Utrecht, Netherlandsm.vermeulen-3@umcutrecht.nl

**Keywords:** DNA methylation, protein-DNA interactions, protein microarray, transcription factors, epigenetics, transcription regulation, Human

## Abstract

A high-throughput screening approach based on a protein microarray reveals that many human transcription factors interact specifically with methylated promoter sequences.

**Related research article** Hu S, Wan J, Su Y, Song Q, Zeng Y, Nguyen HN, Shin J, Cox E, Rho HS, Woodard C, Xia S, Liu S, Lyu H, Ming G-L, Wade H, Song H, Qian J, Zhu H. 2013. DNA methylation presents distinct binding sites for human transcription factors. *eLife* **2**:e00726. doi: 10.7554/eLife.00726

**Image** The transcription factor KLF4 uses specific domains (top) to interact with methylated (bottom) versus non-methylated DNA

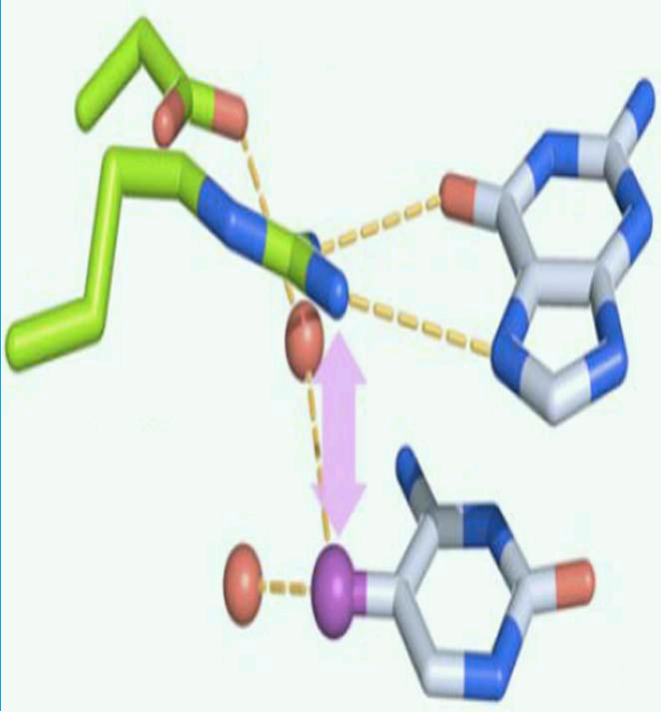

DNA methylation is a key mechanism used by higher eukaryotes to regulate gene expression. The addition of a methyl group to carbon atom number 5 within cytosine bases in DNA is known to repress the transcription of genes into messenger RNA molecules, thus reducing the production of the proteins coded by these genes. Most methylation occurs at CpG dinucleotides—cytosines that are paired with guanines—and these often cluster together to form CpG islands in the promoter regions of genes. In the late 1990s, it was discovered that transcription was repressed when methyl CpG binding proteins were recruited to methylated CpG islands (Hendrich and Bird, 1998).

Subsequent studies have confirmed that the binding of these proteins throughout the genome is proportional to the density of DNA methylation (Baubec et al., 2013), and have identified additional proteins with a high affinity for methylated CpG sites (reviewed in Defossez and Stancheva, 2011). Moreover, in recent years, other screening approaches based mainly on mass spectrometry have revealed that more proteins bind to methylated DNA than previously thought (Mittler et al., 2009; Bartke et al., 2010; Bartels et al., 2011; Spruijt et al., 2013). Now, in *eLife*, Heng Zhu and co-workers at the Johns Hopkins University School of Medicine—including Shaohui Hu as first author—use a high-throughput screening method to show that many human transcription factors also interact with genomic DNA sequences containing methylated CpG sites (Hu et al., 2013).

To this end, the Johns Hopkins researchers made use of a published protein microarray consisting of 1,321 transcription factors and 210 co-factors (Hu et al., 2009). Hu et al. incubated the array with 154 distinct human promoter sequences, each of which contained at least one methylated CpG dinucleotide.Their results revealed that 150 (97%) of the 154 methylated human promoter sequences showed specific binding to at least one protein on the microarray. Moreover, of the 1531 proteins, 47 (3%) showed binding to methylated cytosines within the promoters. Most of the proteins bound to methylated DNA in a sequence-dependent manner; however, a minority bound to many different methylated DNA probes, indicating that binding can sometimes occur independent of DNA sequence (Figure 1).

**Figure 1.**
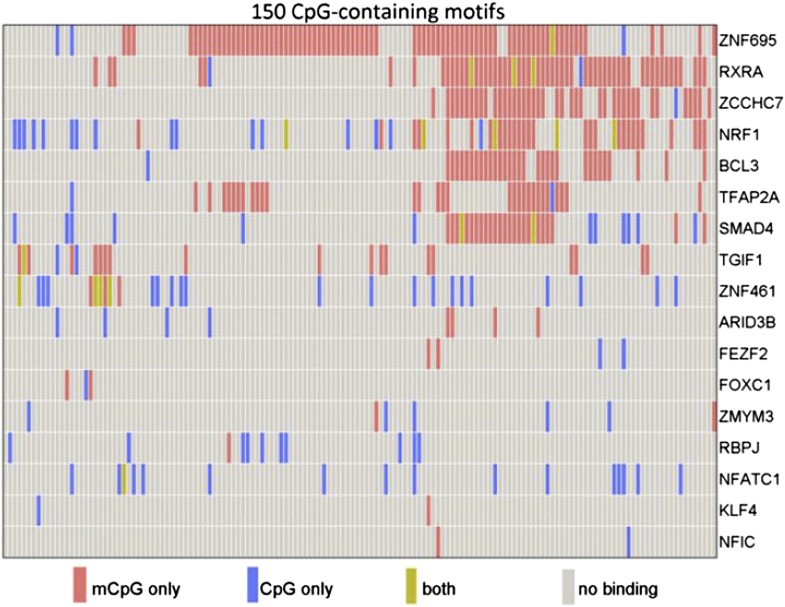
Some human transcription factors can bind to both methylated and non-methylated DNA sequences. Hu et al. examined the ability of 17 human transcription factors to bind to 150 different DNA motifs containing methylated or non-methylated CpG islands. Each row represents one transcription factor. For each motif, some transcription factors bound only to the methylated version (red), some to only the non-methylated version (blue); some to both methylated and non-methylated versions (green), and some to neither (grey). From Figure 2a in Hu et al., 2013.

A number of transcription factors, including KLF4—a recently identified methyl-CpG binding protein (Spruijt et al., 2013)—interacted with methylated sequences that did not resemble their known consensus DNA binding motifs. Using a technique based on electrophoresis, Hu et al. showed that KLF4 binds methylated and non-methylated DNA in a non-competitive manner: this suggests that different domains of the protein may be responsible for each type of binding, which they duly confirmed using molecular modeling and mutagenesis studies.

The Johns Hopkins researchers then mined published ChIP-sequencing data from stem cells to identify the target DNA sequences of KLF4, and compared these with data on genome-wide DNA methylation. Strikingly, KLF4 binding appears to be bimodal in nature throughout the genome, with 38% of KLF4 binding sites showing less than 20% methylation, and 48% showing methylation levels over 80%. Finally, Hu et al. used ChIP-bisulfite sequencing, which makes it possible to determine the methylation status of each cytosine within a target DNA sequence, to confirm that KLF4 also binds to both methylated and non-methylated DNA in vivo.

Hu et al. only profiled a small fraction of the complete human methylome for interactions with transcription factors; further proteins capable of binding genomic methyl CpG sequences surely await identification. The same holds true for interactions with methylated non-CpG sequences such as methyl-CpA (cytosine adjacent to adenine), which are fairly abundant in embryonic stem cells (Ramsahoye et al., 2000; Lister et al., 2009). To determine the physiological relevance of these interactions, it will be important to deduce the affinity with which proteins bind these sequences compared to their known targets; initial experiments along these lines are presented in the current *eLife* paper. Furthermore, recent evidence suggests that non-methylated CpG islands recruit activator proteins, many of which contain a CXXC motif (reviewed in Long et al., 2013). The transcription factor microarray approach used by the Johns Hopkins team, combined with quantitative mass spectrometry-based technology (Spruijt et al., 2013), could thus be used to identify the complete cellular complement of proteins that bind specifically to non-methylated CpG islands.

Finally, this study and other recently published papers force us to reconsider the mechanism(s) via which CpG methylation regulates transcription. Although DNA methylation is generally considered to be a repressive epigenetic modification, experiments presented by Hu et al. suggest that in some cases, methylation of a given promoter sequence can result in activation of transcription. Moreover, other work has revealed a temporal uncoupling of DNA methylation and transcriptional repression during *Xenopus* embryogenesis (Bogdanovic et al., 2011). Further experiments are therefore required to determine whether the functional readout of CpG methylation is affected by the repertoire and abundance of different DNA methylation ‘readers’ acting at any given time in a cell or a developing organism.
